# Influence of HIV and HCV on T cell antigen presentation and challenges in the development of vaccines

**DOI:** 10.3389/fmicb.2014.00514

**Published:** 2014-10-13

**Authors:** Mina John, Silvana Gaudieri

**Affiliations:** ^1^Institute for Immunology and Infectious Diseases, Murdoch UniversityMurdoch, WA, Australia; ^2^Department of Clinical Immunology, PathWest Laboratory Medicine WA, Royal Perth HospitalPerth, WA, Australia; ^3^School of Anatomy, Physiology and Human Biology, University of Western AustraliaCrawley, WA, Australia

**Keywords:** HIV, HCV, viral immune escape, preventative vaccine, anti-viral immune responses

## Abstract

Some of the central challenges for developing effective vaccines against HIV and hepatitis C virus (HCV) are similar. Both infections are caused by small, highly mutable, rapidly replicating RNA viruses with the ability to establish long-term chronic pathogenic infection in human hosts. HIV has caused 60 million infections globally and HCV 180 million and both viruses may co-exist among certain populations by virtue of common blood-borne, sexual, or vertical transmission. Persistence of both pathogens is achieved by evasion of intrinsic, innate, and adaptive immune defenses but with some distinct mechanisms reflecting their differences in evolutionary history, replication characteristics, cell tropism, and visibility to mucosal versus systemic and hepatic immune responses. A potent and durable antibody and T cell response is a likely requirement of future HIV and HCV vaccines. Perhaps the single biggest difference between the two vaccine design challenges is that in HCV, a natural model of protective immunity can be found in those who resolve acute infection spontaneously. Such spontaneous resolvers exhibit durable and functional CD4^+^ and CD8^+^ T cell responses (Diepolder et al., 1995; Cooper et al., 1999; Thimme et al., 2001; Grakoui et al., 2003; Lauer et al., 2004; Schulze Zur Wiesch et al., 2012). However, frequent re-infection suggests partial or lack of protective immunity against heterologous HCV strains, possibly indicative of the degree of genetic diversity of circulating HCV genotypes and subtypes. There is no natural model of protective immunity in HIV, however, studies of “elite controllers,” or individuals who have durably suppressed levels of plasma HIV RNA without antiretroviral therapy, has provided the strongest evidence for CD8^+^ T cell responses in controlling viremia and limiting reservoir burden in established infection. Here we compare and contrast the specific mechanisms of immune evasion used by HIV and HCV, which subvert adaptive human leukocyte antigen (HLA)-restricted T cell immunity in natural infection, and the challenges these pose for designing effective preventative or therapeutic vaccines.

## LEADS FROM GENETIC ASSOCIATION STUDIES SUPPORT IMPORTANCE OF IMMUNOLOGICAL MECHANISMS IN VIRAL INFECTION OUTCOME

Genetic determinants of spontaneous HCV infection clearance and HIV viral control using genome-wide association studies (GWAS) and candidate gene studies have added crucial insight into the influence of the host immune response on infection outcome. For HIV the strongest genetic determinant of viral load set-point and CD4^+^ T cell decline following infection, aside from variants in the CCR5 molecule used by HIV for cell entry, are specific HLA class I alleles (e.g., HLA-B27 and HLA-B57; reviewed in O’Brien et al., 2001) involved in T cell antigen presentation. More recently, a GWAS has shown the association of HLA-C with viral control (International HIVCS et al., 2010). The variation at HLA-C associated with HIV outcome appears to affect cell surface expression of the HLA molecule (Thomas et al., 2009b). The HLA class I molecules also act as ligands for natural killer (NK) cell receptors and this interaction is known to influence the activation threshold for NK cells. Particular combinations of killer immunoglobulin-like receptors (KIR) and HLA class I ligands are strongly associated with HIV infection outcome (Martin et al., 2002).

For HCV, studies that examine host genetic associations with infection outcome clearly indicate that genotypic differences in the interferon pathway such as interferon lambda 3 (IFN-λ3) (Thomas et al., 2009a; Rauch et al., 2010), NK cell cytotoxicity activation threshold (Khakoo et al., 2004) and specific HLA class I and II alleles (McKiernan et al., 2004; Miki et al., 2013) are strongly associated with resolution following HCV infection (reviewed in Rauch et al., 2009a).

For both HIV and HCV, the genetic leads support the involvement of CD8^+^ T cells and antigen presentation in infection outcome. Further evidence can be obtained from the observed heterozygote advantage at the HLA loci for both viral infections (Carrington et al., 1999; Hraber et al., 2007), likely reflecting the presentation of an increased number of T cell targets.

## VIRAL EFFECTS ON ANTIGEN PRESENTATION

To establish chronic infection, viruses such as HIV and HCV must evade the host’s T cell response. The host’s T cell response is governed by the assembly and presentation of antigen via the polymorphic HLA class I and II molecules. In the case of HLA class I presentation of viral peptides to CD8^+^ cytotoxic T cells (CTL), the process requires correct folding of the HLA class I molecules with b2-microglobulin in the endoplasmic reticulum. In parallel, the viral peptides that have been processed by the proteasome complex in the cytosol are then loaded onto the HLA class I-β2-microglobulin complex via the transporter associated with antigen presentation (TAP) protein. This tertiary structure is then translocated to the surface of the cell via the golgi apparatus for presentation to a CTL with the appropriate T cell receptor. Both HIV and HCV have evolved several mechanisms to disrupt this pathway including reduction of HLA class I expression and mutational escape from antigen presentation.

### EFFECTS ON HLA EXPRESSION

For HCV, the proteins core (Miyamoto et al., 2007) and NS3 (Khu et al., 2004) have been shown to affect the function of the proteasome complex (reviewed in Osna, 2009) and potentially HLA class I presentation. Other evidence from the Huh-7 subgenomic replicon system, suggests that HCV infection reduces HLA class I surface expression via a stress-mediated mechanism that lowers the efficacy of HLA class I folding in the endoplasmic reticulum, although the mechanism does not appear to be specific for HLA class I molecules (Tardif and Siddiqui, 2003). However, another study by Herzer et al. (2003) utilizing liver cell lines and plasmid constructs showed increased HLA class I expression via the action of the HCV core protein on TAP1 (function is dependent on p53). Interestingly, increased HLA class I expression was only seen in HepG2 cells (contain functional p53) and not in Huh-7 cells (exhibit a non-functional p53), not for HLA class II (using a pan HLA-DR antibody) and not for other HCV proteins used in a plasmid construct. However, the change in HLA class I expression on the HepG2 cells did not appear to affect CD8^+^ T cell recognition and may instead be related to NK cell cytotoxicity.

The ability to differentiate the effect of HCV on the expression of the different HLA class I loci will be critical given the differing functions/interactions of HLA-A, -B, and -C alleles with NK cell receptors and potentially CD8^+^ T cell antigen presentation. It should be noted that in the studies described above, the pan HLA-class I antibody W6/32 was used and this antibody does not differentiate between the HLA class I loci.

Interactions between HIV and HLA surface expression are well established. HIV Nef in particular down-modulates cell surface expression of HLA-A and -B molecules, rendering them less visible to cytotoxic CD8^+^ T cells, however HLA-C and -E are not selectively down-modulated, which renders them resistant to NK-mediated lysis (Cohen et al., 1999). More recently, differential expression levels of different HLA-C alleles mediated through micro-RNA regulation were found to be important in influencing HIV-1 control. Increased cell surface expression levels of HLA-C were significantly associated with reduced longitudinal viral load and rate of decline in CD4^+^ T cell count in a study involving over 5000 individuals with pre-treatment HIV-1 infection (Apps et al., 2013). Furthermore, this effect was independent of all other HLA allele-specific effects and was robust across different ethnic groups with distinct HLA-C allele frequency distributions and linkage relationships with HLA-A and -B alleles. Further, differential HLA-C expression levels correlated with measured CTL responses and frequency of viral escape mutation, signifying a direct modulatory effect on disease outcome mediated through the quality of HLA-C restricted T cell responses. While this is a “peptide-independent” mechanism of control, it points to the importance of providing sufficient epitopes for HLA-C in a vaccine immunogen not liable to escape from responses binding with high or low expressing HLA-C alleles.

Human leukocyte antigen class II presentation by antigen presenting cells (APCs) to CD4^+^ T cells is important for both HIV and HCV, but less is known about how these viruses affect HLA class II presentation. In general, nascent HLA class II molecules in the endoplasmic reticulum of APCs such as dendritic cells associate with the invariant chain protein, which acts to prevent the binding of endogenous peptides in the HLA class II pocket as well as a chaperone for the HLA class II molecule to the golgi apparatus for transportation to the cell surface. However, cell surface expression of HLA class II molecules is not possible until the invariant chain is degraded by a protease such as cathepsin.

Hepatitis C virus is known to affect dendritic cell function and maturation and has been shown to inhibit HLA class II (HLA-DR) expression on dendritic cells (Siavoshian et al., 2005; Averill et al., 2007; Saito et al., 2008). Subsequent studies have shown that dendritic cells exposed to HCV exhibit decreased expression of Cathepsin S with a corresponding decrease of HLA-DR expression on the cell surface, mainly mediated through the HCV proteins core and NS5A (Kim et al., 2012). Interestingly, hepatocytes may act as APCs in the liver and similar interactions were observed when these cells are transfected with core and NS5A (Kim et al., 2012). More should be examined in this area for HCV as CD4^+^ T cells are critical in HCV infection outcome based on CD4^+^ T cell depletion and HLA class II tetramer studies that clearly show lack of CD4^+^ T cell help and a collapse in HCV-specific CD4^+^ T cell responses within months of acute HCV infection is strongly associated with persistence (Lucas et al., 2007; Schulze Zur Wiesch et al., 2012).

Although less studied compared to interactions with HLA class I, HIV Nef has been shown to influence HLA class II surface expression through effects on intracellular trafficking (Stumptner-Cuvelette et al., 2001). Notably, slower progression of pediatric HIV disease has been associated with *nef* variants, which induced greater down-modulation of surface HLA class II expression, possibly through reducing CD4^+^ T cell activation and therefore cell loss (Schindlera et al., 2007).

### VIRAL ESCAPE, DIVERSITY AND POPULATION LEVEL ADAPTATION

HIV and HCV have error-prone polymerases, rapid replication cycles and in the case of HIV high intracellular recombination rate, allowing for rapid generation, and selective outgrowth of mutant strains, which escape antigen-specific antiviral responses mediated by T cells and NK cells. There is now an extensive literature documenting the predictable mutational networks, which arise in circulating HIV and HCV strains as a result of escape from HLA-restricted T cell responses (Moore et al., 2002; Gaudieri et al., 2006; Rauch et al., 2009b). The antigenic diversity, which partly results from this escape mechanism, is extreme compared to other vaccine-preventable virus infections, and therefore requires especially broad-based immunity from vaccines against HIV and HCV. What makes T cell escape particularly notable is that HLA, which mediates the peptide specific targeting of virally infected cells, is the most polymorphic of human gene systems, having become so as a result of myriad microbial selective pressures in human evolution (Prugnolle et al., 2005). To retain or even increase *in vivo* fitness despite mutation in the context of the great diversity of HLA types across a pandemic infection underscores the plasticity of these viruses and the challenge of vaccinating against them at the population level.

**FIGURE 1:**
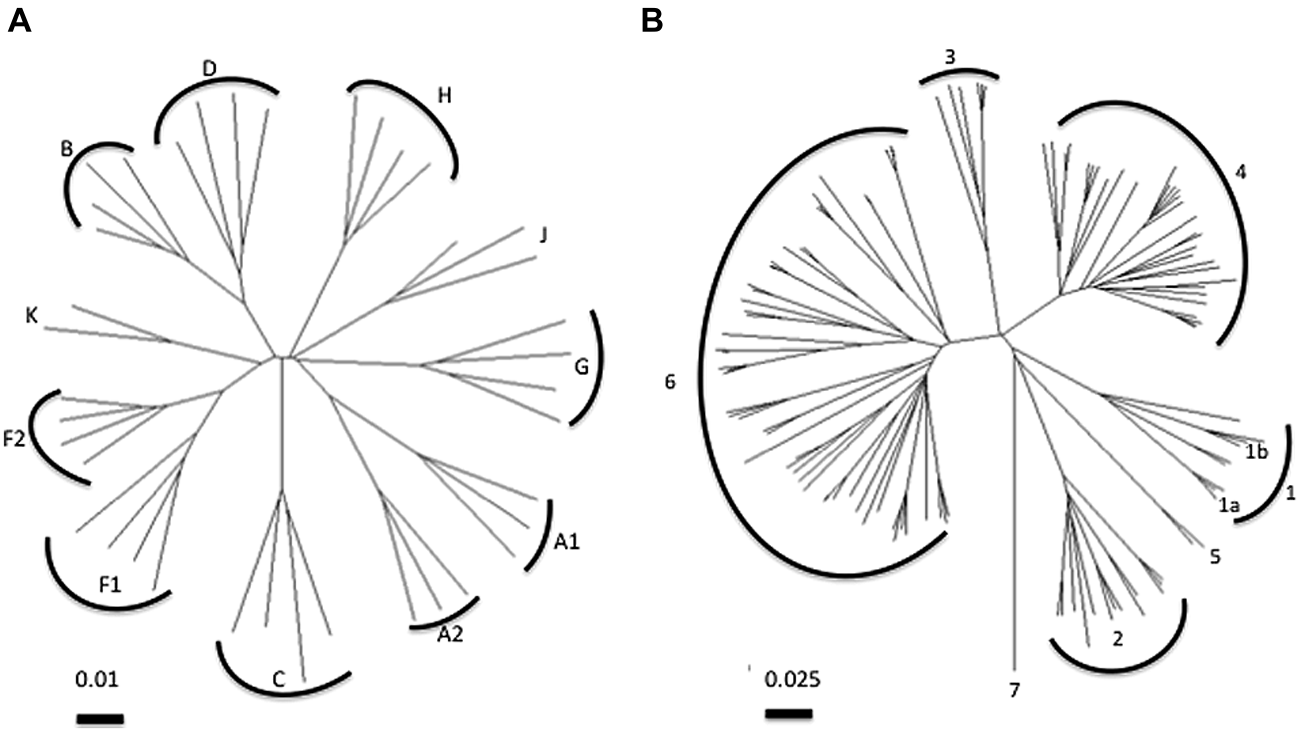
**Phylogenetic analysis of **(A)** HIV pol and **(B)** HCV NS5B polymerase sequences.** Neighbor-joining trees were constructed using the Tamura-Nei model. Note the distance bar for HIV corresponds to 0.01 substitutions per site and for HCV 0.025 substitutions per site. Common HCV subtypes 1a and 1b are indicated on tree. HCV subtype and HIV clade sequences obtained from www.hcv.lanl.gov and www.hiv.lanl.gov,respectively.

In terms of the diversity challenge for vaccines, among the nine phylogenetically distinct HIV-1 group M subtypes, subtypes C and B account for the majority of the global epidemic but have as much as 30–40% inter-subtype diversity at certain segments of the genome. Phylogenetic trees based on HCV sequences indicate the challenge of diversity with HCV, which has an up to 3000-fold higher replication rate than HIV and the absence of any constraint imposed by overlapping open reading frames. HCV genotype 1 is as diverse as all the subtypes of HIV (**Figure 1**). HCV is classified into seven genotypes that differ by about 20–30% at the amino acid level and multiple subtypes for each genotype that differ by 10–15% (Smith et al., 2014). We have previously shown that the polymorphism profile of the different genotypes along sites in the non-structural proteins of HCV vary and supports the observation that there is limited overlap in viral adaptations between genotypes (Rauch et al., 2009b). The limited overlap in the adaptation profile of the genotype 1a and 3a strains likely reflects both different T cell targets as well as different fitness costs associated with variations at specific sites (Salloum et al., 2008; Dazert et al., 2009).

A multi-epitope approach using non-structural proteins has been successful to elicit effective immunity against heterologous HCV strains suggesting potential for effective vaccine development (Folgori et al., 2006; Lang Kuhs et al., 2012). However, a limitation of vaccines developed for HCV is that the use of a small number of T cell epitopes are not sufficient to cover the high variability of HCV observed at the population level (Firbas et al., 2006; Klade et al., 2008). A paucity in the number of known HCV-specific HLA-restricted T cell epitopes is a challenge for a T cell based HCV vaccine.

A further implication of T cell escape is the degree to which escape can accumulate over time in viruses circulating in populations, rendering natural, and vaccine-induced CD8^+^ T cell responses ineffective against transmitting strains, especially those restricted by common HLA alleles. The frequency of certain HLA-driven escape mutations in HIV are highly correlated to HLA allele frequency across ethnically diverse populations, including for some well-known escape networks associated with “protective” HLA alleles (Kawashima et al., 2009). This is an inherently difficult phenomenon to prove, however, as the more such adaptations might accumulate in a population, the less polymorphism and less statistical power to show a correlation with any host trait as evidence of an adaptive process. Notably early population-based studies of HIV and HCV escape detected significant associations between common population HLA alleles and the presence of population consensus amino acids in autologous viruses, which raised the possibility that these were HLA-driven adaptations that had become fixed at the population level (Moore et al., 2002). This clearly has implications for vaccine immunogens, which may include such “population-adapted” areas.

## CURRENT VACCINE DEVELOPMENT

There is recognition of the need to stimulate both arms of the adaptive immune response for an effective preventative HCV vaccine (reviewed in Swadling et al., 2013) and evidence to support the inclusion of both structural and non-structural proteins (reviewed in Torresi et al., 2011). Previous studies have shown evidence of cross-reactive neutralizing antibodies (NAbs), particularly in the chimpanzee model (Choo et al., 1994; Forns et al., 2000; Rollier et al., 2007; Meunier et al., 2011), but limited data on vaccine candidates that elicit both NAbs and HCV-specific T cell responses. Recently, encouraging results have been reported by Martinez-Donato et al. (2014) who utilized a mixture of HCV core, E1, E2, and NS3 in Alum (MixprotHC; containing likely conserved T cell epitopes) from a genotype 1b strain to induce cross-reactive IgG NAbs (to genotype 1a and 2a) and broad HCV-specific CD4^+^ and CD8^+^ T cell responses (detected via proliferation and IFN-gamma ELISpot assays) in immunized mice (BALB/c) and African Green Monkeys. Importantly, immunization with MixprotHC also suppresses viremia in a surrogate challenge model in mice. Other vector-based and DNA-based vaccine candidates exist (reviewed in Swadling et al., 2013) and outcomes from Phase II trials should be informative as to their likely efficacy in “at-risk” populations.

In the comparatively much larger and now 30 year old field of HIV vaccine development, the lack of an effective vaccine points to the many remaining barriers to inducing broadly NAbs or effective CD8^+^ T cells capable of acting and persisting at the site of mucosal HIV entry. Many current vaccine strategies progressing to clinical studies seek to address some of the evasion mechanisms discussed here. For example, there has been testing of various diversity-combating immunogen design approaches, including mosaic vaccines in which inclusion of variant epitopes is optimized, as well as strategies based on conserved immunogens sequences. There are numerous adjuvants, vectors and delivery vehicles designed to improve the efficiency of antigen presentation of vaccine antigens in order to stimulate effective antiviral responses. There are two recent vaccine programs, however, which raise the intriguing possibility that vaccines may need to induce mechanisms of antigen presentation that are highly distinct from those seen in natural infection for their protective effects. A novel “tolerogenic” vaccine consisting of inactivated simian immunodeficiency virus (SIV) mac239 particles with particular bacterial adjuvants has been shown to elicit CD8^+^ T-regulatory cells in vaccinated macaques. These T cell were not cytolytic but were able to suppress the activation of SIV-positive CD4^+^ T cells, rendering them less susceptible to SIV infection after challenge. In addition, these CD8^+^ T cells were found to be uniquely restricted by non-classical MHCIb/E molecules (Andrieu et al., 2014), corresponding to HLA-E in humans. Interestingly, recent data shows HLA-E expression in liver biopsies correlates with HCV viral load in chronic HCV-infected subjects and NK cells lacking the inhibitory receptor for HLA-E (NKG2A) is associated with protection from HCV infection in high-risk exposure subjects (Thoens et al., 2014). To date there has been no examination of non-classical HLA-restricted CD8^+^ T cells in HCV infection. In contrast to the CD8^+^ “T-regulatory” type cells described above, a vaccine based on a rhesis CMV vector has produced durable protection or clearance of SIV challenge infections in vaccinated macaques associated with induction of effector memory CD8^+^ T cell responses. However, these CD8^+^ T cells have been found to target a diverse array of promiscuous or dominant epitopes restricted by HLA class II alleles, rather than HLA class I (Hansen et al., 2013). In both these examples, properties of the vaccine appear to violate the usual rules of CD8^+^ T cell priming and both show promising efficacy in the SIV-macaque model, suggesting novel ways in which vaccines may avoid the evolutionary solutions that SIV/HIV may have developed in natural infection.

## CONCLUSION

In general, induction of CD4^+^ and CD8^+^ T cell responses a key aim of most current vaccine candidates for HIV and HCV, together with innate and humoral immunity as part of a coordinated and long lived immune response. For preventative vaccines, the efficacy of CD4^+^ and CD8^+^ T cells will crucially depend on the extent to which the vaccine induced T cells can overcome natural effects of these viruses on HLA expression, antigen presentation and HLA-associated viral diversity.

## Conflict of Interest Statement

The authors declare that the research was conducted in the absence of any commercial or financial relationships that could be construed as a potential conflict of interest.
